# Serological Assays Based on Recombinant Viral Proteins for the Diagnosis of Arenavirus Hemorrhagic Fevers 

**DOI:** 10.3390/v4102097

**Published:** 2012-10-12

**Authors:** Shuetsu Fukushi, Hideki Tani, Tomoki Yoshikawa, Masayuki Saijo, Shigeru Morikawa

**Affiliations:** Department of Virology, National Institute of Infectious Diseases, 4-7-1 Gakuen, Musashimurayama, Tokyo 208-0011, Japan; Email: htani@nih.go.jp (H.T.); ytomoki@nih.go.jp (T.Y.); msaijo@nih.go.jp (M.S.); morikawa@nih.go.jp (S.M.)

**Keywords:** arenavirus, viral hemorrhagic fever, diagnosis, recombinant protein

## Abstract

The family *Arenaviridae*, genus Arenavirus, consists of two phylogenetically independent groups: Old World (OW) and New World (NW) complexes. The Lassa and Lujo viruses in the OW complex and the Guanarito, Junin, Machupo, Sabia, and Chapare viruses in the NW complex cause viral hemorrhagic fever (VHF) in humans, leading to serious public health concerns. These viruses are also considered potential bioterrorism agents. Therefore, it is of great importance to detect these pathogens rapidly and specifically in order to minimize the risk and scale of arenavirus outbreaks. However, these arenaviruses are classified as BSL-4 pathogens, thus making it difficult to develop diagnostic techniques for these virus infections in institutes without BSL-4 facilities. To overcome these difficulties, antibody detection systems in the form of an enzyme-linked immunosorbent assay (ELISA) and an indirect immunofluorescence assay were developed using recombinant nucleoproteins (rNPs) derived from these viruses. Furthermore, several antigen-detection assays were developed. For example, novel monoclonal antibodies (mAbs) to the rNPs of Lassa and Junin viruses were generated. Sandwich antigen-capture (Ag-capture) ELISAs using these mAbs as capture antibodies were developed and confirmed to be sensitive and specific for detecting the respective arenavirus NPs. These rNP-based assays were proposed to be useful not only for an etiological diagnosis of VHFs, but also for seroepidemiological studies on VHFs. We recently developed arenavirus neutralization assays using vesicular stomatitis virus (VSV)-based pseudotypes bearing arenavirus recombinant glycoproteins. The goal of this article is to review the recent advances in developing laboratory diagnostic assays based on recombinant viral proteins for the diagnosis of VHFs and epidemiological studies on the VHFs caused by arenaviruses.

## 1. Introduction

The virus family *Arenaviridae* consists of only one genus, but most viruses within this genus can be divided into two different groups: the Old World arenaviruses and the New World arenaviruses (also known as the Tacaribe complex) [1,2]. The differences between the two groups have been established through the use of serological assays. Most of the arenaviruses cause persistent infection in rodents without any symptoms, and humans acquire a variety of diseases when zoonotically infected. Lymphocytic choriomeningitis virus (LCMV) is the only arenavirus to exhibit a worldwide distribution, and causes illnesses such as meningitis [3,4]. Congenital LCMV infections have also been reported [4,5]. Most importantly, viral hemorrhagic fever (VHF) can be caused by several arenaviruses. Lassa fever, caused by the Lassa virus (LASV), an Old World arenavirus, is one of the most devastating VHFs in humans [6]. Hemorrhaging and organ failure occur in a subset of patients infected with this virus, and it is associated with high mortality. Many cases of Lassa fever occur in Western Africa in countries such as Guinea, Sierra Leone, and Nigeria [7,8,9,10,11,12,13]. Tacaribe complex lineage B of the New World arenaviruses consists of the Junin virus (JUNV), Guanarito virus (GUNV), Sabia virus (SABV) and Machupo virus (MACV), the etiological agents of Argentine, Venezuelan, Brazilian, and Bolivian hemorrhagic fevers, respectively [14,15]. Although genetically distinct from one another, they appear to produce similar symptoms, accompanied by hemorrhaging in humans [14,15]. These pathogenic New World arenavirus species are closely associated with a specific rodent species [6].

Humans are usually infected with pathogenic arenaviruses through direct contact with tissue or blood, or after inhaling aerosolized particles from urine, feces, and saliva of infected rodents. After an incubation period of 1–3 weeks, infected individuals abruptly develop fever, retrosternal pain, sore throat, back pain, cough, abdominal pain, vomiting, diarrhea, conjunctivitis, facial swelling, proteinuria, and mucosal bleeding. Neurological problems have also been described, including hearing loss, tremors, and encephalitis. Because the symptoms of pathogenic arenavirus-related illness are varied and nonspecific, the clinical diagnosis is often difficult [14,16]. Human-to-human transmission may occur via mucosal or cutaneous contact, or through nosocomial contamination [14,16]. These viruses are also considered to be potential bioterrorism agents [2].

A number of arenavirus species have been recently discovered as a result of both rodent surveys and disease outbreaks [17,18,19,20,21,22,23,24,25,26]. A novel pathogenic New World arenavirus, Chapare virus (CHPV), has been isolated from a fatal case of VHF in Bolivia [20]. In addition, five cases of VHF have been reported in South Africa, and a novel arenavirus, named Lujo virus, was isolated from a patient [17]. The Lujo virus is most distantly related to the other Old World arenaviruses [17]. To date, there is no information concerning the vertebrate host for the Chapare and Lujo viruses.

There is some evidence of endemicity of the Lassa virus in neighboring countries [27,28]. However, as the magnitude of international trade and travel is continuously increasing, and the perturbation of the environment (due either to human activity or natural ecological changes) may result in behavioral changes of reservoir rodents, highly pathogenic arenaviruses could be introduced to virus-free countries from endemic areas. In fact, more than twenty cases of Lassa fever have been reported outside of the endemic region in areas such as the USA, Canada, Europe, and Japan [29,30,31,32,33]. It is of great importance to detect these pathogens rapidly and specifically in order to minimize the risk and scale of outbreaks of VHFs caused by arenaviruses. However, these arenaviruses are classified as biosafety level (BSL)-4 pathogens, making it difficult to develop diagnostic techniques for these virus infections in laboratories without BSL-4 facilities. To overcome these difficulties, we have established recombinant viral nucleoproteins (rNPs)-based serological assays, such as IgG-enzyme-linked immunosorbent assay (ELISA), indirect immunofluorescence assay (IFA), and antigen (Ag)-capture ELISA for the diagnosis of VHFs caused by highly pathogenic arenaviruses. Furthermore, virus neutralization assays using pseudotype virus-bearing arenavirus GPs have been developed. In this review, we describe the usefulness of such recombinant protein-based diagnostic assays for diagnosing VHFs caused by arenaviruses. 

## 2. Currently Used Diagnostic Techniques for VHFs

In outbreaks of VHFs, infections are confirmed by various laboratory diagnostic methods. Virus detection is performed by virus isolation, reverse transcription-polymerase chain reaction (RT-PCR), and antigen-capture ELISA. It has been shown that monoclonal antibody panels against pathogenic arenaviruses are useful for detecting viral antigens on the virus-infected cells as well as for investigating of antigenic relationships of arenaviruses [34,35,36]. Detection of the virus genome is suitable for a rapid and sensitive diagnosis of VHF patients in the early stage of illness, and extensive reviews of such RT-PCR assays have been described [37,38]. More recently, progress in the RT-PCR method covering genetic variations of the hemorrhagic fever viruses (HFVs) [39,40] and a multiplexed oligonucleotide microarray for the differential diagnosis of VHFs have also been reported [41]. On the other hand, antibodies against these viruses can be detected by the indirect immunofluorescence assay (IFA), or IgG- and IgM-ELISA. An IFA detects the antibody in the serum, which is able to bind to the fixed monolayer of the virus-infected cells. Although the interpretation of immunofluorescence results requires experience, the assay has advantages over other methods, since each virus generates a characteristic fluorescence pattern that adds specificity to the assay compared to a simple ELISA readout. A serological diagnosis by the detection of specific IgM and IgG antibodies to the HFVs must be sensitive, specific and reliable, because a misdiagnosis can lead to panic in the general population. An IgM-specific ELISA is suitable for detecting recent infection, but the relevance of IgM testing for acute VHF depends on the virus and the duration of illness; specific IgM is not often present in the very early stage of illness, and patients who die of VHF often fail to seroconvert at all. An IgG-specific ELISA is efficacious, not only in the diagnosis of a large number of VHF cases, especially during convalescence, but also for epidemiological studies in the endemic regions. The detailed methods used for the IFA and IgG- and IgM-ELISAs for the diagnosis of VHF using authentic virus-antigens have been described in detail [42,43,44,45].

## 3. Recombinant Protein-Based ELISA for Detecting Antibodies against Arenaviruses

Arenaviruses have a bisegmented, negative-sense, single stranded RNA genome with a unique ambisense coding strategy that produces just four known proteins: a glycoprotein, a nucleoprotein (NP), a matrix protein (Z), and a polymerase (L) [46]. Of these proteins, the NP is the most abundant in virus-infected cells. Recombinant protein technology could meet the demand for a simple and reliable VHF test system, and recombinant NP (rNP) has been shown to be useful for serological surveys of IgM- and IgG antibodies against arenaviruses [47,48,49,50]. 

### 3.1. Antibody Detection-ELISA

**Figure 1:**
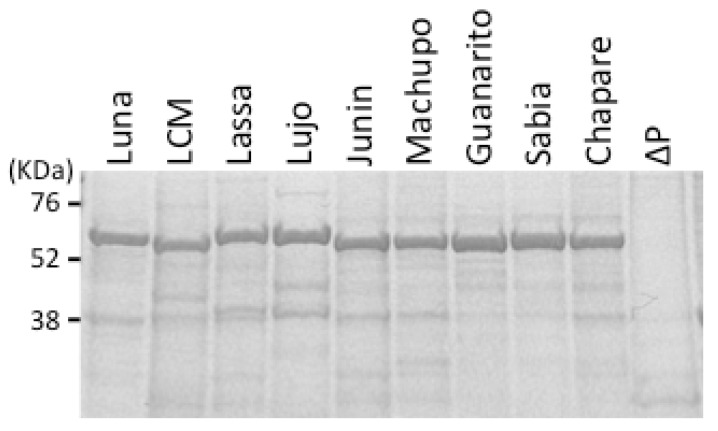
Purified rNPs. The expression and purification efficiency of arenavirus rNP were analyzed by sodium dodecyl sulfate-polyacrylamide gel electrophoresis (SDS-PAGE) after staining the gels with Coomassie blue. Purified NP antigens with approximate molecular weights of 62 kDa from Luna, LCM, Lassa, Lujo, Junin, Machupo, Guanarito, Sabia, and Chapare viruses and the purified negative control antigen (ΔP) are shown.

Recombinant baculoviruses that express the full-length rNP of arenaviruses have been generated [48,50,51]. The method used for the purification of arenavirus rNP from insect *Tn5* cells infected with recombinant baculoviruses is effective and simple compared to those for Ebola, Marburg, and Crimean-Congo hemorrhagic fever virus rNPs [51,52,53,54,55]. Most of the arenavirus rNPs expressed in insect cells using the recombinant baculoviruses are crystallized [56] and are solubilized in PBS containing 8M urea. Since the majority of *Tn5* cellular proteins are solubilized in PBS containing 2M urea, the arenavirus rNPs in the insoluble fraction in PBS containing 2M urea can be solubilized by sonication in PBS containing 8M urea. After a simple centrifugation of the lysates in PBS containing 8M urea, the supernatant fractions can be used as purified rNP antigens without further purification steps [51]. The control antigen is produced from *Tn5* cells infected with baculovirus lacking the polyhedrin gene (ΔP) in the same manner as the arenavirus rNPs (Figure 1). 

As described above, recombinant baculoviruses allow the delivery of rNP antigens without using infectious live arenaviruses. An ELISA plate coated with the predetermined optimal quantity of purified rNPs (approximately 100 ng/well) is used for the IgG-antibody detection assay. An advantage of using recombinant rNP for the IgG-ELISA is that it enables a direct comparison of antibody cross-reactivity among arenavirus rNPs, since antigen preparations of all arenavirus rNPs tested are performed using the same method [51]. Rabbit anti-sera raised against LCMV-rNP and LASV-rNP show cross-reactivity to LASV-rNP and LCMV-rNP, respectively, indicating that rabbit antibodies against rNPs of Old World arenaviruses cross-react with rNPs of other Old World arenaviruses (Table 1) [51]. Similarly, rabbit anti-sera generated against JUNV-NP show cross-reactivity to the LASV-rNP and LCMV-rNP, although the reaction is weak. However, rabbit anti-sera against LASV-NP and LCMV-NP show a negative reaction to the JUNV-rNP (Table 1) [51], indicating that rabbit antibodies against JUNV (a pathogenic New World arenavirus) NP might cross-react with the Old World arenavirus NP, whereas antibodies against Old World arenavirus NPs may not be able to react with pathogenic New World arenavirus NPs.

The rNP-based IgG-ELISA has also been used for the characterization of a mouse monoclonal antibody (MAb). Nakauchi *et al.* [50] have investigated the cross-reactivity of MAbs against JUNV rNP to pathogenic New World arenavirus rNPs, as well as LASV rNP. MAb C11-12 reacts at the same level with the rNPs of all of the pathogenic New World arenaviruses, including JUNV, GTOV, MACV, SABV, and CHPV, indicating that this MAb recognizes an epitope conserved among pathogenic New World arenaviruses. Another MAb, C6-9, reacts specifically with the rNP of JUNV, but does not react with those of the other pathogenic New World arenaviruses [50]. This indicates that MAb C6-9 recognizes a JUNV-specific epitope. None of these MAbs reacts with the rNP of the human pathogenic Old World arenavirus LASV. Thus, the MAb C11-12 is considered to be a broadly reactive MAb against New World arenaviruses, whereas MAb C6-9 is JUNV-specific. These findings have been confirmed by detailed epitope analyses using peptide mapping [50]. Similarly, the cross-reactivity of MAbs against LASV rNP has been analyzed [51]. MAb 4A5 cross-reacts with the Mopeia virus (MOPV) but not with the LCMV rNP. MAb 6C11 cross-reacts with LCMV rNP, while MAb 2-11 does not cross-react with LCMV rNP [51].

**Table 1:** Anti-serum reactivity for rNPs of different arenaviruses in IgG ELISAs.

Rabbit anti-serum	Reactivity for rNP from		
	LASV	LCMV	JUNV
anti-LASV NP	**++**	**+**	**−**
anti-LCMV NP	**+**	**++**	**−**
anti-JUNV NP	**+**	**+**	**++**

It is important to evaluate whether rNP-based ELISA is useful for the diagnosis of human VHF cases. The specificity of the LASV-rNP-based IgG ELISA has been confirmed by using sera obtained from Lassa fever patients [51]. The Lassa fever patients’ sera show a highly positive reaction in the LASV-rNP-based IgG-ELISA, but sera from patients with Argentine hemorrhagic fever (AHF), which is caused by JUNV, do not. The serum from an AHF patient showed a highly positive reaction in the JUNV-rNP-based IgG-ELISA [49]. In addition, it was shown that, using sera obtained from AHF cases, the results of the JUNV rNP-based IgG ELISA correlate well with an authentic JUNV antigen-based IgG ELISA [49]. An IgM-capture ELISA using purified LASV-rNP as an antigen has been developed in the same way as in previous reports [54,57] and detects an LASV-IgM antibody [58]. In addition, immunoblot assays based on N-terminally truncated LASV rNP have been developed for detecting IgG and IgM antibodies against LASV. These methods may provide a rapid and simple Lassa fever test for use under field conditions [47].

### 3.2. Antibody Detection IFA

An IFA using virus-infected cells is a common antibody test for VHF viruses [59,60,61,62,63]. To avoid the use of highly pathogenic viruses for the antigen preparation, mammalian cells expressing recombinant rNP have been developed [51,57,64,65,66,67,68]. Lassa virus NP antigen for IFA can be prepared simply as described [51]. Briefly, the procedure involves (1) transfecting HeLa cells with a mammalian cell expression vector inserted with the cloned NP cDNA; (2) expanding the stable NP-expressing cells by antibiotic selection; (3) mixing the rNP-expressing cells with un-transfected HeLa cells (at a ratio of 1:1); (4) spotting the cell mixtures onto glass slides, then drying and fixing them in acetone.

**Figure 2:**
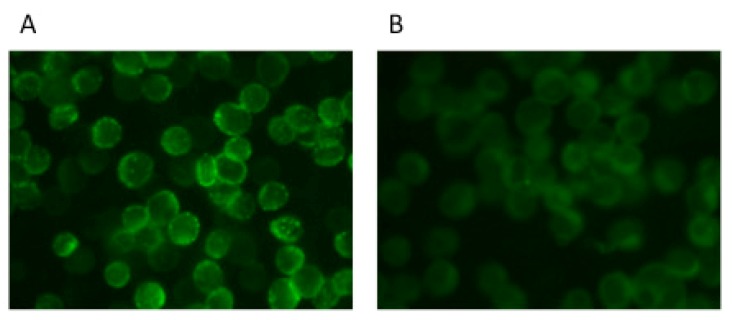
Staining patterns of the LASV-rNP-expressing HeLa cells obtained from the sera of a Lassa-NP-immunized monkey (**A**) and control serum (**B**) in an IFA.

In the IFA specific for LASV-NP, antibody positive sera show characteristic granular staining patterns in the cytoplasm (Figure 2) [69], thus making it easy to distinguish positive from negative samples. The specificity of the assay has also been confirmed by using sera obtained from Lassa fever patients [51]. In addition, an IFA using JUNV rNP-expressing HeLa cells has been developed to detect antibodies against JUNV, and the assay has been evaluated by using AHF patients’ sera [70]. The LASV-rNP-based antibody detection systems such as ELISA and IFA are suggested to be useful not only for the diagnosis of Lassa fever, but also for seroepidemiological studies of LASV infection. In our preliminary study, approximately 15% of the sera collected from 334 Ghanaians and less than 3% of 280 Zambians showed positive reactions in the LASV-rNP-based IgG ELISA [58]. These results are in agreement with the fact that Lassa fever is endemic to the West African region, including Ghana, but less in the East African region.

## 4. Antigen-Capture ELISA

For the diagnosis of many viral infections, PCR assays have been shown to have an excellent analytical sensitivity, but the established techniques are limited by their requirement for expensive equipment and technical expertise. Moreover, the high degree of genetic variability of the RNA viruses, including arenavirus and bunyavirus, poses difficulties in selecting primers for RT-PCR assays that can detect all strains of the virus. Since the sensitivity of the Ag-capture ELISA is comparable to that of RT-PCR for several virus-mediated infectious diseases, including Lassa fever and filovirus hemorrhagic fever [51,71,72,73], the Ag-capture ELISA is a sophisticated approach that can be used for the diagnosis of viral infections. Ag-capture ELISAs detecting viral NP in viremic sera have been widely applied to detect various viruses, since they are the most abundant viral antigens and have highly conserved amino acid sequences [50,51,54,71,72,74,75]. Polyclonal anti-sera or a mixture of MAbs present in the ascetic fluids from animals immunized for HFVs have been used for capture-antibodies in the Ag-capture ELISA [36,76,77,78,79]. MAbs recognizing conserved epitopes of the rNP are also used as capture antibodies since they have a high specificity for the antigens, and an identification of the epitopes of these MAbs is of crucial importance for the assessment of the specificity and cross-reactivity of the assay system [50,51,53,54,71,75]. In order to develop a sensitive diagnostic test for Lassa fever and AHF, rNPs of LASV and JUNV (see above) have been prepared, and newly established MAbs against them have been characterized and used for Ag-capture ELISAs [50,51]. The Ag-capture ELISA using MAb 4A5 has been confirmed to be useful in the detection of authentic LASV antigen in sera serially collected from hamsters infected with LASV [51]. The sensitivity of the MAb 4A5-based Ag-capture ELISA was similar to that of conventional RT-PCR, suggesting that the Ag-capture ELISA can be efficiently used in the diagnosis of Lassa fever [51]. Therefore, the MAb 4A5- based Ag-capture ELISA is considered to be useful in the diagnosis of Lassa fever. Also, by using MAbs raised against the rNP of JUNV, Ag-capture ELISAs specific for JUNV and broadly reactive to human pathogenic New World arenaviruses have been developed [50]. The Ag-capture ELISA using MAb E4-2 and C11-12 detected the Ags of all of the pathogenic New World arenaviruses tested, including JUNV. On the other hand, the Ag-capture ELISA using MAb C6-9 detects only the JUNV Ag. Considering that the symptoms of JUNV infection in humans are indistinguishable from those due to other pathogenic New World arenaviruses, the Ag capture ELISA using MAb C6-9 may be a useful diagnostic tool, especially for AHF [50]. 

## 5. Neutralization Assays Based on VSV Pseudotypes

The virus neutralization assay is accepted as the “gold standard” serodiagnostic assay to quantify the antibody response to infection and vaccination of a wide variety of viruses associated with human diseases [80,81,82,83,84,85,86]. The presence of neutralizing antibodies is a reliable indicator of protective immunity against VHF [87,88,89]. The most direct method for detection of neutralizing antibodies against HFVs is by plaque reduction neutralization tests using infectious viruses. However, because of the high pathogenicity of HFVs to humans and the strict regulation of select agents, only a limited number of laboratories are able to perform such neutralization tests. For many HFVs, replication-incompetent pseudotyped virus particles bearing viral envelope protein (GP) have been shown to mimic the respective HFV infections, thus, neutralization assays using the pseudotypes may be advantageous in some laboratory settings for the detection of antibodies to HFVs without the need for heightened biocontainment requirements. 

**Figure 3:**
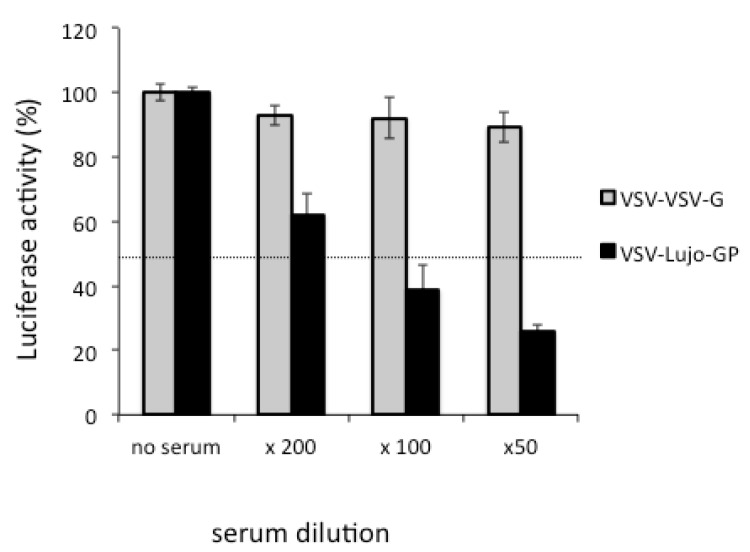
Neutralization assay for VSV-Lujo-GP. VSV-Lujo-GP or a control pseudotype (VSV-VSV-G) that expressed luciferase was incubated with serially diluted serum obtained from a rabbit immunized with Lujo-GPC, and was then inoculated in triplicate into Vero E6 cells. The luciferase activity (%) of each well compared to the negative control (no serum) is shown.

The VSV-based vector has already been used to generate replication-competent recombinant VSVs to study of the role of GPs of various viruses [90,91,92]. Recent advances in producing pseudotype virus particles have enabled the investigation of the virus cell entry, viral tropism, and effect of entry inhibitors, as well as measurement of the neutralization titers, by using human immunodeficiency virus-, feline immunodeficiency virus-, murine leukemia virus-, or VSV-based vectors [86,93,94,95,96,97,98,99,100,101,102,103]. Pseudotypes based on VSV have advantages compared with other pseudotypes based on retroviruses for the following reasons. First, the pseudotype virus titer obtained with the VSV system is generally higher than that of the pseudotyped retrovirus system [104]. Second, the infection of target cells with a VSV pseudotype can be readily detected as green fluorescent protein (GFP)-positive cells at 7–16 h post-infection because of the high level of GFP expression in the VSV system [104,105]. In contrast, the time required for infection in the pseudotyped retrovirus system is 48 h [106,107], which is similar to the time required for infectious viruses to replicate to a level that results in plaque-forming or cytopathic effects in infected cells. A high-throughput assay for determining neutralizing antibody titers using VSV pseudotypes expressing secreted alkaline phosphatase [108,109] or luciferase (Figure 3) has also been developed.

**Figure 4:**
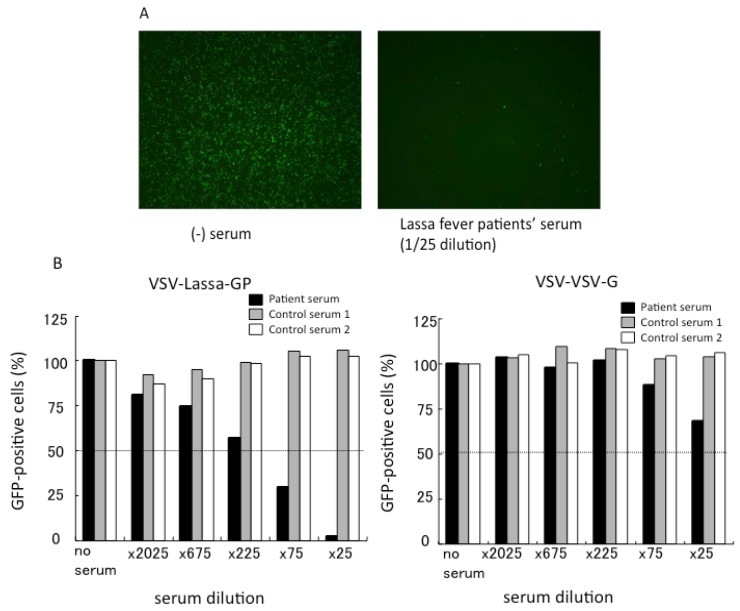
Neutralization assay for VSV-Lassa-GP. (**A**) VSV-LAS-GP was incubated with or without serum obtained from a Lassa patient, and then was inoculated into Vero cells. The GFP signal was observed under a fluorescence microscope. (**B**) VSV-LAS-GP or the control pseudotype (VSV-VSV-G) incubated with serially diluted patient serum or healthy control sera were inoculated into Vero E6 cells. The relative number of GFP-positive cells (%) compared with negative control cells (no serum) is shown.

We have recently developed a VSV-based pseudotype bearing Lassa virus GP (VSV-LAS-GP) for the detection of neutralizing antibodies in the sera obtained from a Lassa fever patient. An example of the LASV neutralization assay using the VSV pseudotype is shown (Figure 4). In the presence of serum from Lassa fever patients, the number of GFP-positive cells (infectivity of VSV-LAS-GP) is significantly reduced compared with the number in the absence of the patient’s serum (Figure 4A). The control VSV pseudotype bearing VSV GP (VSV-VSV-G) is not neutralized by any sera. When the cut‑off serum dilution is set at 50% inhibition of infectivity compared with the infectivity in the absence of the test serum, the neutralization titer of this patient’s serum for VSV-LAS-GP is calculated to be 75 (Figure 4B). Likewise, a VSV-based pseudotype bearing the Junin virus GP has been developed for the detection of neutralizing antibodies from AHF patients’ sera. The accuracy of the results of VSV-based neutralization assays has been confirmed by comparison with the results of the neutralization assay using live Junin virus [70].

The Lujo virus is a new member of the hemorrhagic fever-associated arenavirus family from Zambia and southern Africa, and the virus is classified as a BSL-4 pathogen [17]. The genome sequence analysis of the Lujo virus suggests that the virus is genetically distinct from previously characterized arenaviruses. In order to study the infectivity of this newly identified arenavirus, we have recently developed a luciferase-expressing VSV pseudotype bearing Lujo virus GPC (VSV-Lujo-GP). As shown in Figure 3, infection with VSV-Lujo-GPC is specifically neutralized by rabbit anti-Lujo GPC serum. Thus, the VSV-Lujo-GP may be a useful tool not only for determining the neutralizing antibody titer within the serum, but also for exploring yet-to-be-defined cellular receptor(s) for Lujo virus infection or for screening inhibitors of the Lujo virus GP-mediated cell entry.

## 6. Conclusions

Hemorrhagic fever outbreaks caused by pathogenic arenaviruses result in high fatality rates. A rapid and accurate diagnosis is a critical first step in any outbreak. Serologic diagnostic methods for VHFs most often employ an ELISA, IFA, and/or virus neutralization assay. Diagnostic methods using recombinant viral proteins have been developed and their utilities for diagnosing of VHF have been reviewed. IgG- and IgM- ELISAs and IFAs using rNPs as antigens are useful for the detection of antibodies induced in the patients’ sera. These methods are also useful for seroepidemiological surveys for HFVs. Ag-capture ELISAs using MAbs to the arenavirus rNPs are specific for the virus species or can be broadly reactive for New World arenaviruses, depending on the MAb used. Furthermore, the VSV-based pseudotype system provides a safe and rapid tool for measuring virus neutralizing antibody titers, as well as a model to analyze the entry of the respective arenavirus in susceptible cells without using live arenaviruses. Recent discoveries of novel arenavirus species [17,26,110] and their potential to evolve predominantly via host switching, rather than with their hosts [110,111], suggest that an unknown pathogenic arenavirus may emerge in the future, and that the diagnostic methods for VHF caused by arenaviruses should thus be further developed and improved.
